# Retraction: Urban transportation system toughness assessment under New Crown epidemics

**DOI:** 10.1371/journal.pone.0324224

**Published:** 2025-05-12

**Authors:** The PLOS One Editors

The *PLOS One* Editors retract this article [1] due to concerns about:

- Compliance with PLOS policies on Manipulation of the Publication Process and Artificial Intelligence Tools and Technologies.
- Compromised peer review.
- The use of terminology that differs from standards in the field (e.g., “New Crown Epidemics” instead of “COVID-19 Pandemic”).
- The reliability of the reported results and conclusions.

All authors either did not respond directly or could not be reached.
